# Lymphocytes and related inflammatory factors as predictors of metabolic syndrome risk in shift workers: A machine learning approach based on large-scale population data

**DOI:** 10.1371/journal.pone.0339673

**Published:** 2025-12-29

**Authors:** Yiren Bao, Rui Wang

**Affiliations:** Department of massage, The Hangzhou TCM Hospital Affiliated to Zhejiang Chinese Medical University, Hangzhou, Zhejiang, China; University of Montenegro-Faculty of Medicine, MONTENEGRO

## Abstract

**Background:**

Metabolic syndrome (MetS) is characterized by chronic inflammation and can be worsened by circadian disruption, which is common among shift work. Machine learning can predict the risk of MetS in shift workers using inflammatory biomarkers. Most investigations have focused on the general population rather than shift workers, a distinct group that requires continuous health monitoring; therefore, we aimed to examine the relationship between inflammatory indicators and MetS using blood cell counts in this high-risk group of shift workers who require long-term health monitoring and to enhance the biological understanding of MetS by applying machine learning methods.

**Methods:**

In this cross-sectional study, we analyzed data from shift workers included in the National Health and Nutrition Examination Survey between 2005–2010 and 2017–2018. Prediction models, including random forest (RF), extreme gradient boosting (XGBoost), light gradient boosting machine (LightGBM), and linear regression (LR), were developed and validated. We compared the model performance and conducted stratified analysis, smooth curve fitting, and threshold effect analysis to further explore the relationship between inflammation and MetS risk in shift workers.

**Results:**

The analysis included 3,079 participants in total. Each machine learning model demonstrated good predictive performance in assessing MetS risk among shift workers. LightGBM achieved the area under the curve (AUC) of 0.944 in training dataset and 0.722 in testing dataset; XGBoost had an AUC of 0.818 in training dataset and 0.747 in testing dataset; and LR had an AUC of 0.763 in training dataset and 0.699 in testing dataset, RF had an AUC of 0.741 in training dataset and 0.729 in testing dataset.

Furthermore, the analysis revealed that body mass index, age, neutrophil, lymphocyte, monocyte, and platelet counts, along with their derived inflammatory indices, were significant predictors. Multivariate logistic regression adjusted for lifestyle and health factors showed that lymphocytes remained consistently associated with MetS in shift workers. Generalized additive model analysis revealed complex non-linear relationships between lymphocytes and platelets. Inflammatory factors strongly predicted MetS risk in shift workers, with their effects varying by concentration threshold, particularly for lymphocytes (k = 2.2, right side p < 0.001).

**Conclusion:**

Lymphocyte counts and related composite indices are significant predictors of MetS risk in shift workers. Consistent monitoring of these biomarkers may be useful for early odds-based stratification of MetS in this high-risk population, whereas any preventive implications would require confirmation in longitudinal and interventional studies.

## Introduction

Metabolic syndrome (MetS) is characterized by a cluster of metabolic abnormalities and cardiovascular risk factors, including central obesity, hypertriglyceridemia, low high-density lipoprotein cholesterol, hyperglycemia, and high blood pressure. Diagnosis requires the fulfillment of at least three of the five criteria [1]. An unhealthy diet and/or lifestyle contribute to the development of MetS, affecting approximately one-quarter of the global population, with prevalence increasing with age [2].

However, prevalence rates vary across countries. For instance, the estimated prevalence of MetS is 33.4% in the United States, 28.8% in Turkey, and 14.4% in China [3–5]. MetS significantly increases the risk of cardiovascular disease, cancer, and chronic kidney disease, significant causes of morbidity and mortality worldwide [6–8]. Because MetS imposes significant health and economic burdens, identifying new risk factors and improving early prevention remain key priorities for researchers in related fields.

The influence of work-related stress factors on MetS remains unclear. Shift workers face unique challenges, particularly irregular working schedules and night shifts. With rapid industrial development, shift work has become increasingly common worldwide. Approximately 15–30% of the population in Europe and the United States engage in shift work across various occupations, and this proportion continues to rise. This percentage is similarly high in China.

Disruptions in circadian rhythms negatively affect sleep quality, fatigue levels, and emotional well-being and may also accelerate the onset of MetS, stroke, and cancer [9]. The body’s internal biological clock regulates the sleep-wake cycle, metabolism, and hormone secretion. Shift work forces a misalignment between circadian rhythms and environmental cues, resulting in circadian rhythm disorders that disrupt the timing and efficiency of metabolic processes. These disruptions can lead to glucose intolerance, weight gain, and lipid metabolism disorders [10], which are key components of MetS.

Inflammation often coexists in patients with MetS and circadian rhythm disorders, suggesting a complex interaction among these conditions. Chronic inflammation is consistently linked to the pathophysiology of MetS. Inflammatory markers worsen insulin resistance and impair endothelial function. Additionally, they promote fat accumulation and increase the risk of cardiovascular complications [11]. Previous cross-sectional studies have primarily concentrated on the role of inflammatory factors and their derived indices in metabolic disorders [12,13].

Moreover, persistent low-grade inflammation is common among shift-workers and acts as a significant trigger for circadian rhythm disorders [14,15]. As a high-risk group for circadian disruption, shift workers exhibit inflammatory responses that remain insufficiently characterized, and comprehensive analyses of inflammation at different levels in MetS are lacking.

Current research has examined the association between inflammation and circadian rhythm disruptions related to MetS, relying mainly on traditional statistical methods. For instance, Li et al. conducted an observational study using correlation analysis, linear regression, and receiver operating characteristic (ROC) curve calculations to investigate whether disruptions in the circadian clock contributed to MetS components in patients with obstructive sleep apnea [16]. Similarly, Romero-Cabrera et al. conducted a prospective randomized controlled trial lasting up to 4 years, carefully selecting intervention methods and assessment criteria and applying statistical analyses such as analysis of variance (ANOVA) and linear mixed-effects models for repeated measures [17].

However, these traditional methods often require large datasets, involve multiple assumptions, and have strict application criteria.

In contrast, machine learning (ML) provides a robust approach for computer-assisted data mining and analysis and excels at identifying complex patterns and nonlinear relationships within data. This methodology is widely applied in fields such as engineering and medicine, particularly for developing predictive tools [18,19]. Several studies have used ML algorithms to predict the risk of MetS based on physical examination findings or genetic information. Li et al. developed a super learner model that integrates multiple ML algorithms using physical examination variables, such as vital signs, serum indicators, and comorbidities, to predict MetS risk [20]. Similarly, Huang et al. extracted genomic deoxyribonucleic acid (DNA) from serum and applied genetic risk score models based on ML to predict the occurrence of MetS [21]. However, most of these investigations have focused on the general population rather than shift workers, a distinct group that requires continuous health monitoring.

In this study, we used data from the National Health and Nutrition Examination Survey (NHANES) to develop an ML prediction model for assessing the risk of MetS among shift workers. The primary objectives are twofold: (1) to examine the relationship between inflammatory indicators and MetS using blood cell counts in this high-risk group of shift workers who require long-term health monitoring and (2) to enhance the biological understanding of MetS by applying ML methods.

## Methods

### Study population and design

The study population was derived from the NHANES, a comprehensive survey that collects nationally representative health and nutritional data from participants in the United States. Extensive household interviews were conducted to obtain demographic and health history information. Physical examinations and blood sample collections were performed in a Mobile Examination Center (MEC). Serum samples were analyzed by the Laboratory Sciences Division of the National Center for Environmental Health at the Centers for Disease Control and Prevention.

This cross-sectional study used publicly available data from NHANES for the years 2005–2010 and 2017–2018 and was approved by the National Center for Health Statistics Research Ethics Review Board. Further details are available on the official NHANES website (https://www.cdc.gov/nchs/nhanes/about_nhanes.htm). The original study protocol and formal approval from the Research Ethics Review Board (protocol #2005−06; #2011−17) can be accessed on the NHANES Research Ethics Review Board website (https://www.cdc.gov/nchs/nhanes/irba98.htm). All participants provided written informed consent at the time of registration. Our analysis included 3,079 employed individuals aged 20 years or older who completed both interviews and MEC assessments. Participants were required to have a shift work schedule to be included in the study. We excluded pregnant women and multiracial individuals to minimize potential bias. Pregnancy induces significant physiological changes that can affect MetS assessment. Similarly, participants from different racial backgrounds were excluded because MetS risk factors can vary by ethnicity. As the MetS definition used in this study is primarily suited to European and American populations, we focused on individuals from these groups to maintain consistency.

### Assessment of shift work schedule

Daytime work was defined as standard weekday work between 9:00 a.m. and 5:00 p.m. Shift work included any schedule that differed from these regular daytime hours [22]. Shift work status was determined using questionnaire items such as: “Which best describes your hours worked?” and “What was your overall work schedule for the past 3 months?” The response options included “evening or nights, “early mornings,” or “variable schedule (early mornings, days, and nights).” For analysis, participants who reported standard daytime work were classified as the “Regular” group, and those who reported any nonstandard schedule were classified as “Irregular.”

### Assessment of MetS

MetS was defined according to the harmonized criteria proposed in the joint interim statement of the International Diabetes Federation Task Force on Epidemiology and Prevention, National Heart, Lung, and Blood Institute, American Heart Association, World Heart Federation, International Atherosclerosis Society, and the International Association for the Study of Obesity [23]. Participants were diagnosed with MetS if they met three or more of the following criteria: (1) waist circumference: ≥ 102 cm in men or ≥ 88 cm in women; (2) triglycerides: ≥ 150 mg/dL or current use of lipid-lowering medication; (3) High-density lipoprotein cholesterol (HDL-C): HDL-C < 40 mg/dL for men or < 50 mg/dL for women; (4) Blood pressure: systolic ≥ 130 mmHg and/or diastolic ≥ 85 mmHg, or current use of antihypertensive medication; and (5) Fasting glucose: elevated levels or current use of antihyperglycemic agents.

### Assessment of inflammation

Fasting venous blood samples were collected from all participants to measure leukocyte, neutrophil, lymphocyte, monocyte, and platelet counts, expressed as thousands of cells per microliter (1000 cells/μL). The following complete blood count (CBC)-derived inflammatory indicators were calculated: Systemic Immune-Inflammation Index (SII), Systemic Inflammatory Response Index (SIRI), Systemic Inflammatory Composite Index (AISI), Monocyte-to-Lymphocyte Ratio (MLR), Neutrophil-to-Monocyte Ratio (NMLR), Platelet-to-Lymphocyte ratio (PLR), and Neutrophil-to-Lymphocyte Ratio (NLR) [24]. The formulas were defined as follows:


SII = (neutrophil count × platelet count)/ lymphocyte count

SIRI = (neutrophil count × monocyte count)/ lymphocyte count

AISI = (neutrophil count × monocyte count × platelet count)/ lymphocyte count

MLR, monocyte count/ lymphocyte count

NMLR = (neutrophil count + monocyte count)/ lymphocyte count

NLR = neutrophil count/ lymphocyte count

PLR = platelet count/ lymphocyte count

### Covariates

In this study, we examined several sociodemographic factors, including age, sex, and race (non-Hispanic Black, non-Hispanic white, Mexican American and other Hispanic). Education level was characterized as less than high school, high school diploma/GED, or college and above. The poverty-income ratio (PIR) and alcohol consumption (non-drinker, 1–5 drinks/month, 5–10 drinks/month, or ≥10 drinks/month) were also assessed. Smoking status was classified as never smoked, former smoker, or current smoker. Body mass index (BMI) and self-reported diet quality (excellent, very good, good, fair, or poor) were included.

Dietary quality was further evaluated using the Healthy Eating Index-2020 (HEI-2020), which measures adherence to the Dietary Guidelines for Americans based on 13 food and nutrient components. Furthermore, we considered health complications (including weak/failing kidneys, stroke, asthma, anemia, arthritis, congestive heart failure, coronary heart disease, chronic bronchitis, cancer, or malignancy) and Patient Health Questionnaire (PHQ) scores. These covariates were selected based on previous research [25,26].

### Variable selection and model construction

Four standard ML models were developed using the shift worker dataset, which served as the model development cohort. A total of 32 variables collected in this study were included in the modeling process. The models applied in this analysis were random forest (RF), extreme gradient boosting (XGBoost), light gradient boosting machine (LightGBM), and linear regression (LR).

The dataset was randomly divided into training and test sets following the widely accepted Pareto principle, with a 70:30 split. Specifically, 70% of the data was used for model selection and tuning, while the remaining 30% was reserved for evaluating performance. During the training, 10-fold cross-validation was employed to ensure robust model validation. In this procedure, the training set was divided into 10 subsets: nine were used to train the model, and one served as the validation set. This process was repeated 10 times so that each subset was used once for validation. The algorithm’s final accuracy was then calculated as the average across the 10 iterations. Following this validation process, the test set (30% of the data) was used to evaluate how well the model generalized to unseen data.

The analytic sample was randomly split into a training dataset accounting for 70% and an independent test dataset accounting for 30%. The training dataset was used for model development and internal validation, and the test dataset was reserved solely for evaluating generalization to unseen data. For all ML algorithms, performance within the training dataset was assessed using cross-validated out-of-fold predictions, or out-of-bag (OOB) predictions for RF. The primary performance metric was AUC.

For LR, XGBoost, and LightGBM, we implemented 10-fold cross-validation using the training dataset. In each iteration, nine folds were used to fit the model and the remaining fold served as the validation fold, so that every observation was predicted only from models that had not been trained on it. Hyperparameters for the XGBoost and LightGBM, such as learning rate, maximum depth, and the number of boosting iterations, were tuned to maximize cross-validated AUC [27]. The LR model was fitted without penalization as a baseline parametric classifier and did not require extensive hyperparameter tuning. After cross-validation, each algorithm was refitted on the full training dataset using the optimal hyperparameters, and its generalization performance was evaluated on the independent 30% test dataset.

The RF model was implemented using bootstrap aggregation with OOB estimation. Each tree was grown on a bootstrap sample of approximately 80% of the training observations, with the remaining observations serving as OOB samples for that tree. For each participant, the OOB prediction was obtained by aggregating votes from trees that did not include that participant in their bootstrap sample, providing an internal cross-validation. To reduce overfitting, we restricted tree complexity by using 1,000 trees, sampling 80% of observations per tree, setting the number of variables considered at each split to the square root of the total number of predictors, and enforcing a minimum terminal node size of 5 observations [28,29].

All models were implemented using the “RandomForest,” “lightgbm,” “treeshap,” “xgboost,” and “fastshap” packages.

### Model validation and explainability

To thoroughly evaluate model performance, we utilized standard binary classification metrics, including area under the curve (AUC), accuracy (ACC), sensitivity (recall), and specificity. Furthermore, we analyzed the ROC curve to gain further insight into model behavior. The bootstrap method was used to refine estimates of model performance.

A major limitation of complex ML models is their inherent lack of interpretability, often referred to as the “black box” nature [30,31]. To enhance interpretability, we used SHapley Additive ExPlanations (SHAP) values from the ‘shap’ package, which are based on game theory, to visualize feature importance. This approach enhances model transparency and interpretability, thereby improving the reliability of its predictions [32].

In our study, we utilized the permutation importance method to identify the most significant features for predicting MetS in shift workers. This method evaluates feature importance by measuring how randomly shuffling a feature’s values affects the model’s predictive performance. To ensure reliability and minimize errors, we conducted 1,000 permutations for each feature across all models, yielding importance values that were averaged and ranked to determine their relative contributions. This methodology enables researchers and practitioners to better understand the factors influencing model outcomes by analyzing feature importance, recognizing interdependencies, and considering key elements for informed decision-making [30].

### Statistical analysis

Continuous variables were presented as mean, median, minimum, and maximum values, whereas categorical variables were expressed as frequencies and percentages. Participants were divided into MetS and non-MetS groups according to diagnostic criteria. Differences between the two groups were analyzed using the Wilcoxon rank-sum or Kruskal–Wallis test for continuous variables and the Fisher exact or chi-squared test for categorical variables.

Missing data were addressed using multiple imputation, a flexible, simulation-based statistical method commonly applied to handle missing values or nonresponse in the NHANES dataset [33]. To ensure nationally representative estimates, analyses were weighted per NHANES guidelines.

Logistic regression analysis was used to examine the correlation between inflammatory markers and MetS, with results expressed as odds ratios (OR) and 95% confidence intervals (95% CIs). Given that the relationships between inflammatory indicators and MetS may vary among participants due to multiple influencing factors, we conducted stratified analyses to assess these differences in participants with MetS. We also applied a generalized additive model (GAM) to explore potential non-linear relationships. When a non-linear association was detected, a two-piecewise linear regression model was constructed to calculate the threshold effect of inflammation levels on MetS using the resulting smoothing plots. When the smoothing curve revealed a relationship between MetS status and inflammation levels, a recursive method was used to automatically identify the inflection point for subsequent analyses.

All statistical analyses were performed using the R software (http://www.r-project.org) and EmpowerStats (http://www.empowerstats.com), with statistical significance defined as p < 0.05.

## Results

### Participant characteristics

**Fig 1:**
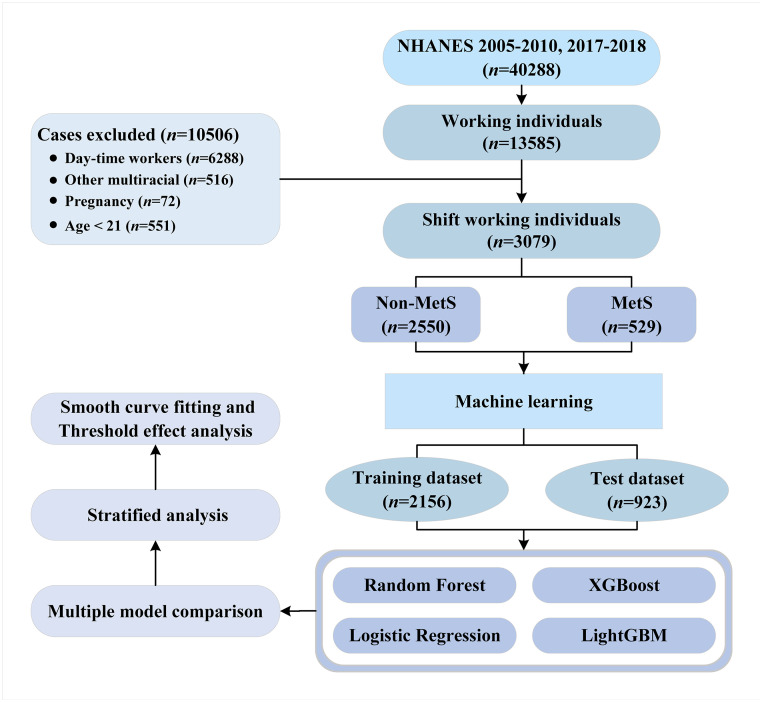
Flow chart of participant selection and study design. NHANES: National health and nutrition examination survey; MetS: Metabolic syndrome; XGBoost: eXtreme gradient boosting; LightGBM: Light gradient boosting machine.

We screened the shift worker population and included 3,079 individuals in the study cohort. Participants were divided into two groups: MetS (unweighted n = 529, weighted n = 9,291,315) and non-MetS (unweighted n = 2,250, weighted n = 26,216,750). A detailed flowchart of the selection process is presented in Fig 1. Table 1 shows the baseline characteristics of the two groups. Participants with MetS were generally older (median age: 45 vs. 38, p < 0.001), more likely to be overweight (34.2% vs. 30.5%) or obese (61.6% vs. 32.2%), and exhibited higher levels of inflammatory markers, including neutrophils (median: 4.00 vs. 3.60, p < 0.001), lymphocytes (median: 2.10 vs. 2.00, p = 0.024), monocytes (median: 0.60 vs. 0.50, p = 0.009), SIRI (median: 0.91 vs. 1.02, p = 0.048) and AISI (median: 223 vs. 246, p = 0.027). No significant differences were observed in shift work patterns or other inflammatory indicators between the groups.

**Table 1:** Weighted Patient demographics and baseline characteristics.

Characteristic	Non MetS Weighted N = 26,216,750 Unweighted n = 2,550^1^	MetS Weighted N = 9,291,315 Unweighted n = 529^1^	p-value
**Shift schedule**			0.488^2^
** irregular**	56.1%	53.2%	
** regular**	43.9%	46.8%	
**Age**	38 (29, 48)	45 (38, 56)	< 0.001^3^
**Sex**			0.021^2^
** male**	55.7%	66.0%	
** female**	44.3%	34.0%	
**Race**			0.324^2^
** Other Hispanic**	7.5%	6.1%	
** Non-Hispanic White**	66.5%	66.0%	
** Non-Hispanic Black**	15.3%	13.9%	
** Mexican American**	10.7%	13.9%	
**Education**			0.487^2^
** High school diploma/ GED**	11.9%	13.3%	
** Less than high school**	6.6%	8.3%	
** Some college or above**	81.5%	78.4%	
**PIR**	2.65 (1.70, 4.62)	2.65 (1.64, 4.26)	0.790^3^
**Alcohol drinking**			0.866^2^
** Non-drinker**	51.4%	53.6%	
** 1–5 drinks/ month**	32.4%	30.7%	
** 5–10 drinks/ month**	7.5%	6.2%	
** 10 + drinks/ month**	8.7%	9.5%	
**Smoke**			0.555^2^
** Never smoker**	54.8%	51.6%	
** Former smoker**	22.1%	26.1%	
** Current smoker**	23.1%	22.3%	
**BMI**			< 0.001^2^
** Underweight (< 18.5)**	1.5%	0.1%	
** Normal (18.5 to < 25)**	35.8%	4.1%	
** Overweight (25 to < 30)**	30.5%	34.2%	
** Obese (30 or greater)**	32.2%	61.6%	
**Self-diet report**			0.456^2^
** Excellent**	1.8%	2.1%	
** Very good**	7.5%	4.1%	
** Good**	80.8%	81.9%	
** Fair**	7.6%	9.2%	
** Poor**	2.3%	2.8%	
**HEI2020**	48 (42, 54)	48 (42, 52)	0.570^3^
**Weak/ failing kidneys**			0.126^2^
** No**	99.0%	98.1%	
** Yes**	1.0%	1.9%	
**Stroke**			0.250^2^
** No**	99.2%	98.3%	
** Yes**	0.8%	1.7%	
**Asthma**			0.174^2^
** No**	84.9%	88.5%	
** Yes**	15.1%	11.5%	
**Anemia**			0.868^2^
** No**	97.6%	97.4%	
** Yes**	2.4%	2.6%	
**Arthritis**			< 0.001^2^
** No**	86.0%	74.1%	
** Yes**	14.0%	25.9%	
**Congestive heart failure**			0.180^2^
** No**	99.0%	97.7%	
** Yes**	1.0%	2.3%	
**Coronary heart disease**			0.343^2^
** No**	98.3%	97.1%	
** Yes**	1.7%	2.9%	
**Chronic bronchitis**			0.198^2^
** No**	95.6%	93.6%	
** Yes**	4.4%	6.4%	
**Cancer or malignancy**			0.238^2^
** No**	94.5%	92.0%	
** Yes**	5.5%	8.0%	
**PHQ score**	1.00 (1.00, 3.00)	1.00 (1.00, 4.00)	0.630^3^
**Neutrophils**	3.60 (2.90, 4.70)	4.00 (3.10, 4.90)	< 0.001^3^
**Lymphocytes**	2.00 (1.60, 2.40)	2.10 (1.70, 2.60)	0.024^3^
**Monocytes**	0.50 (0.40, 0.60)	0.60 (0.50, 0.70)	0.009^3^
**Platelets**	240 (207, 277)	243 (207, 287)	0.480^3^
**SII**	421 (312, 625)	431 (312, 645)	0.474^3^
**SIRI**	0.91 (0.67, 1.38)	1.02 (0.70, 1.45)	0.048^3^
**AISI**	223 (147, 347)	246 (165, 381)	0.027^3^
**MLR**	0.26 (0.21, 0.33)	0.26 (0.21, 0.32)	0.767^3^
**NMLR**	2.00 (1.62, 2.74)	2.04 (1.53, 2.82)	0.940^3^
**NLR**	1.77 (1.39, 2.42)	1.77 (1.34, 2.54)	0.876^3^
**PLR**	121 (96, 149)	116 (89, 142)	0.129^3^

^1^%; Median (Q1, Q3)

^2^Pearson’s X^2: Rao & Scott adjustment

^3^Design-based Kruskal–Wallis test

### Development and validation of prediction models

Of all participants, 2,166 were randomly assigned to the training set and 913 to the test set. All variables were included in training the four ML models, and the AUC was used to evaluate their discriminative performance.

**Fig 2:**
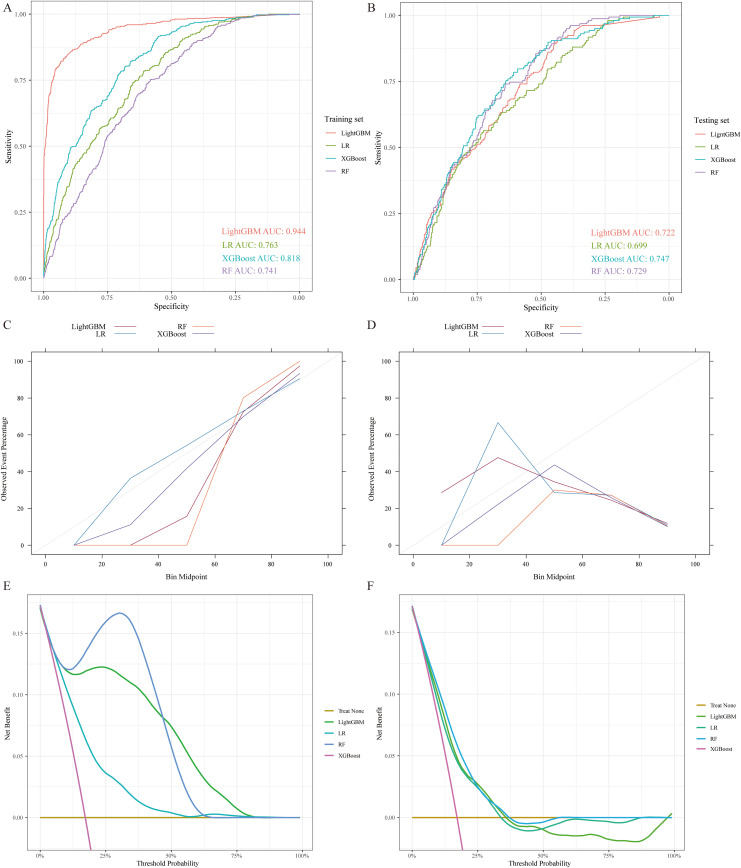
Model performance comparison. **A.** ROC curves of the four algorithms on the training set; **B.** ROC curves on the test set; **C.** Calibration curve for the training set; **D.** Calibration curve for the test set; **E.** DCA curve for the training set; **F.** DCA curve for the test set. ROC curve**s**: comparing the discriminative ability of the four algorithms in the training and test sets, with the AUC representing overall classification performance. **Calibration curve**s illustrate the agreement between predicted and observed probabilities, the closer the curve is to the 45° diagonal, the better the calibration. **DCA curve**: illustrating the net clinical benefit of each model across a range of threshold probabilities.

In the LightGBM model (Fig 2A, red line), the AUC for the training dataset was 0.944, which was considerably higher than that for the test dataset (Fig 2B, red line; 0.722). In comparison, the RF model demonstrated an AUC of 0.741 in the training dataset (Fig 2A, purple line) and 0.729 in the test dataset (Fig 2B, purple line). Similarly, the XGBoost model achieved an AUC of 0.818 for the training dataset (Fig 2A, blue line) and 0.747 for the test dataset (Fig 2B, blue line). LR yielded AUC values, 0.763 for the training dataset (Fig 2A, green line) and 0.699 for the test dataset (Fig 2B, green line). All models showed some degree of performance decline when applied to the test dataset; however, the decrease was less significant in RF and XGBoost than in LightGBM. Evaluation of calibration (Fig 2C) and DCA (Fig 2E) for all four models on the training set confirmed that LR (Fig 2C, blue line; Fig 2E, blue-green line) performed acceptable. followed closely by XGBoost (Figs 2C and 2E, purple line), LightGBM (Figs 2C and 2E, brown and green lines), and RF (Fig 2C and 2E, orange and blue line). When evaluated on the test dataset (Figs 2D and 2F), XGBoost (Figs 2D and 2F, purple line) was the best-performing algorithms, followed closely by LR (Figs 2D and 2F, blue and blue-green line), LightGBM (Figs 2D and 2F, brown and green lines), and RF (Figs 2D and 2F, orange and blue line). Per-model performance metrics, including AUC values with 95% CIs estimated via bootstrap resampling, as well as sensitivity, specificity, and calibration data at clinically relevant thresholds, are provided in the S1 and S2 Table.

**Fig 3:**
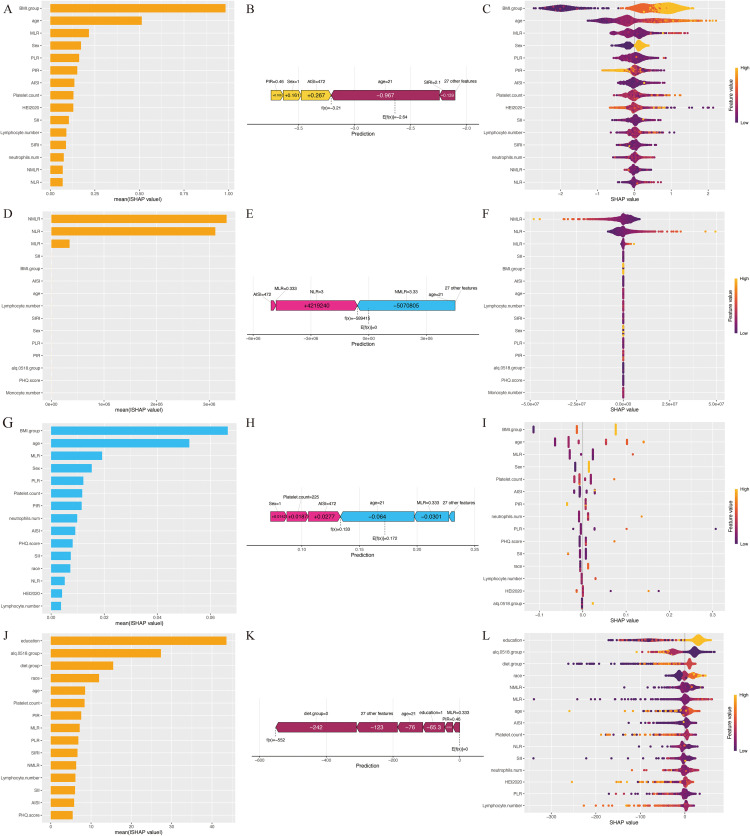
Lightgbm, LR, RF and XGBoost algorithm for identifying essential features. A: Bar plot of feature importance based on lightgbm. B: SHAP force plot on lightgbm. C: Beeswarm plot of effects of features based on lightgbm-shap. D: Bar plot of feature importance based on LR. E: SHAP force plot on LR. F: Beeswarm plot of effects of features based on LR-shap. G: Bar plot of feature importance based on RF. H: SHAP force plot on RF. I: Beeswarm plot of effects of features based on RF-shap. J: Bar plot of feature importance based on Xgboost. K: SHAP force plot on Xgboost. L: Beeswarm plot of effects of features based on Xgboost -shap. **Bar plots**: showing the ranked feature importance scores for each algorithm. **SHAP force plots**: displaying the individual contribution of each feature to model predictions. **SHAP beeswarm plots**: summarizing the distribution and magnitude of feature effects, where color represents feature value and position indicates the SHAP value (positive or negative influence on prediction).

The bar plot highlights the importance of various features across four models: LightGBM, LR, XGBoost, and RF. In the LightGBM model, the top 15 features identified include BMI, age, sex, and several inflammatory ratios such as MLR, PLR, AISI, SII, SIRI, NMLR and NLR, among others (Fig 3A). Fig 3B compares individual incidence risk (f(x) = −3,21) with the overall average risk (E[f(x)] = −2.64), showing that the individual has a lower risk than the population mean. Factors such as PIR, sex, and AISI contribute to this elevated risk.

The beeswarm plot presents the overall importance of features in the LightGBM, LR, XGBoost, and RF models. A color scale on the right indicates the relative value of each feature, where yellow dots represent high values and purple dots indicate low values. The violin plot, aligned at the midline, aggregates the dots representing each case in the internal validation set; the vertical spread reflects the number of cases sharing identical SHAP values. Fig 3C displays the SHAP values of the 15 features and their association with MetS risk among shift workers in the LightGBM model. Higher values of BMI, age, MLR, and platelet counts were associated with increased risk, while higher values of PLR, PIR, SII, SIRI, and other inflammatory markers were associated with decreased risk.

Figs 3D and 3F present the top 15 features contributing to predictions in the LR model, Figs 3G and 3I show the top 15 predictive features in the XGBoost model, and Figs 3J and 3L depict the top 15 influential features identified by the RF model. The findings indicate that, in addition to established risk factors for MetS such as BMI and age, inflammatory predictors, including lymphocytes, neutrophils, monocytes, and platelets, are also significant [34,35]. Moreover, previous studies have reported associations between these inflammatory factors and both shift work and MetS [15,36]. The risk contribution of each feature closely mirrors that observed in the LightGBM model. When comparing individual and overall incidence risks, the LR model (Fig 3E, f(x) = −589415, E[f(x)] = 0), the XGBoost model (Fig 3H, f(x) = 0.133, E[f(x)] = 0.172), and the RF model (Fig 3K, f(x) = −552, E[f(x)] = 0) all demonstrated lower individual risks than the overall average risk. Across all models, the individual risk consistently falls below the overall risk.

**Fig 4:**
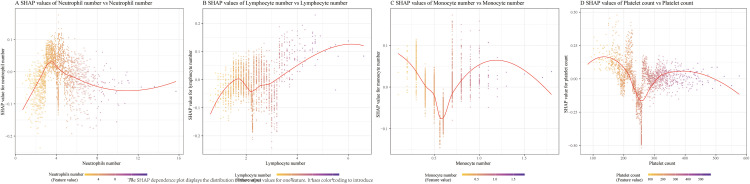
The relationship among the hub feature’s levels and SHAP values. A: SHAP values of neutrophils. B: SHAP values of lymphocytes. C: SHAP values of monocytes. D: SHAP values of platelets. Scatter plots show how SHAP values vary with neutrophil, lymphocyte, monocyte, and platelet counts, respectively, reflecting each feature’s influence on model output.

The relationship between hub feature levels and SHAP values (Fig 4.) showed that the varying trends of different inflammatory factors influenced the model’s predictive performance. The SHAP value of neutrophils increased sharply with neutrophil count (Fig 4A), peaking within the range of 3.5–3.7, and then exhibited a U-shaped trend, first decreasing and then increasing, with the lowest value observed within the range of 10–12. The SHAP value of lymphocytes (Fig 4B) showed an overall stable upward trend, with a slight U-shaped pattern within the range of 1.6–2.5. In contrast, the SHAP value of monocytes (Fig 4C) showed an opposite trend to that of neutrophils, decreasing sharply to its lowest point (0.6–0.7), followed by an inverted U-shaped trend. The SHAP value of platelets (Fig 4D) exhibited two inverted U-shaped patterns: the first occurred within the range of 100–250, reaching a trough around 250–260, followed by the second inverted U-shaped trend. Platelet counts within the range of approximately 220–300 and > 500 indicated a negative contribution to the model predictions.

To facilitate interpretation of feature effects and potential interactions, SHAP dependence plots were generated exclusively for the LR model. Unlike tree-based models such as LightGBM, RF, and XGBoost, which can capture complex non-linear relationships [29,31], LR offers a more transparent and interpretable framework for visualizing the relationship between individual predictors and model outputs. The inherently linear structure of LR enables clearer identification of both the direction and magnitude of feature contributions, as well as more interpretable interactions via color-coded modifiers in SHAP plots. In the LR model, age and lymphocyte count exhibited a positive and approximately linear association with SHAP values, while sex emerged as a prominent interaction factor. In contrast to non-linear models that often produce unstable SHAP dependence plots, the LR model provides more stable and interpretable visualizations. This clarity makes LR particularly valuable for examining feature relationships in analyses based on SHAP values [28].

**Fig 5:**
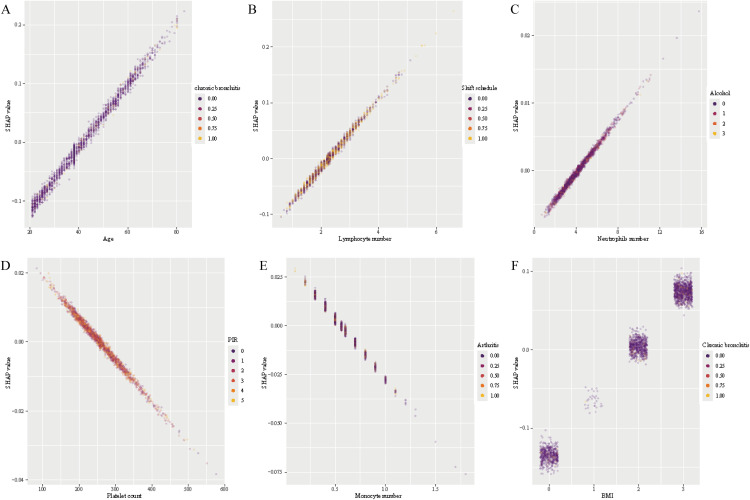
SHAP feature dependence plots on LR. A: SHAP feature dependence plot (Age & Chronic bronchitis). B: SHAP feature dependence plot (Lymphocytes & Shift schedule). C: SHAP feature dependence plot (Neutrophils & Alcohol). D: SHAP feature dependence plot (Platelets & PIR). E: SHAP feature dependence plot (Monocytes & Arthritis). F: SHAP feature dependence plot (BMI & Chronic bronchitis). Each plot illustrates the interaction between two variables, where the x-axis represents the feature value and the y-axis represents the SHAP value, indicating the strength and direction of each feature’s contribution to the model prediction.

The SHAP dependence plot displays the distribution of output values for a single feature and uses color coding to introduce a second feature, enhancing the analysis of potential feature interactions. In the LR model (Fig 5.), age (Fig 5A), and lymphocyte count (Fig 5B) were positively and linearly correlated with SHAP values. Chronic bronchitis and shift schedule were used for color classification; however, their effects on the SHAP values of age and lymphocytes were relatively low. Neutrophils (Fig 5C) also demonstrated a positive linear correlation with SHAP values, with alcohol consumption as an interaction factor significantly influencing the SHAP values. Conversely, platelet count (Fig 5D) exhibited a negative linear correlation with SHAP values, and PIR, as an interaction factor, significantly influenced SHAP values. Monocyte counts (Fig 5E) were negatively correlated with the model predictions. Among the categorical variables, participants who were overweight or obese demonstrated significantly higher SHAP values than those in other groups (Fig 5F), suggesting that the interaction between BMI positively influenced model predictions.

### Association between inflammation factors and MetS in shift workers

Different models were used to adjust for covariates in the multivariate logistic regression analyses examining the association between inflammatory factors and MetS risk (Table 2). For neutrophils, Models I (unadjusted) and II (adjusted for age, sex, and race) showed a significant positive association with MetS risk; however, this association was no longer significant in Model III (further adjusted for shift schedule, PIR, alcohol consumption, smoking, BMI, self-reported diet quality, HEI2020, weak/failing kidneys, stroke, asthma, anemia, arthritis, coronary heart disease, chronic bronchitis, cancer or malignancy, and PHQ score). Similarly, lymphocytes remained consistently associated with elevated MetS risk across all models, with the strongest associations observed in Models II and III, suggesting their potential as reliable predictors. In Model I, monocytes were significantly associated with increased risk. However, the association became non-significant in Model III, indicating that additional covariates may mediate this relationship. When analyzed categorically, participants in the third quartile (Q3: 0.563–0.6) had significantly lower odds of MetS across all models (Model I: OR: 0.02; 95% CI: 0.00–0.08, p < 0.001; Model II: OR: 0.02; 95% CI: 0.00–0.09, p < 0.001; Model III: OR: 0.01, 95% CI: 0.00–0.06, p < 0.001) compared with the reference group. For platelet count, no significant associations were observed in any model (all ORs = 1.00, all p > 0.05). However, in the categorical analysis using quartiles, significant associations were observed for Q3 [255–287) across all models (Model I: OR: 0.53; 95% CI: 0.40–0.70, p < 0.001; Model II: OR: 0.65; 95% CI: 0.49–0.87, p = 0.004; Model III: OR: 0.56; 95% CI: 0.41–0.75, p < 0.001). Additional analyses of other inflammatory markers are presented in S3 Table.

**Table 2:** Multivariate logistic regression analysis of different inflammation factors.

Characteristic	Model I			Model II			Model III		
	OR^1^	95% CI^1^	p-value	OR^1^	95% CI^1^	p-value	OR^1^	95% CI^1^	p-value
**Neutrophils (continuous)**	1.09	1.03, 1.16	0.002	1.10	1.04, 1.17	0.002	1.05	0.98, 1.12	0.184
**Neutrophils**									
** Q1 [0.7,3.1)**	—	—		—	—		—	—	
** Q2 [3.1,4.1)**	1.35	1.03, 1.78	0.033	1.31	0.99, 1.75	0.062	1.13	0.83, 1.53	0.435
** Q3 [4.1,4.9)**	1.00	0.75, 1.32	0.976	1.02	0.76, 1.37	0.887	0.76	0.55, 1.04	0.081
** Q4 [4.9,15.7]**	1.50	1.15, 1.96	0.003	1.52	1.15, 2.02	0.004	1.11	0.82, 1.50	0.509
** p for trend**			0.031			0.026			0.947
**Lymphocytes (continuous)**	1.21	1.06, 1.38	0.006	1.31	1.14, 1.50	< 0.001	1.18	1.02, 1.36	0.025
**Lymphocytes**									
** Q1 [0.6,1.8)**	—	—		—	—		—	—	
** Q2 [1.8,2.25)**	1.25	0.96, 1.63	0.100	1.32	1.00, 1.74	0.049	1.26	0.94, 1.68	0.124
** Q3 [2.25,2.6)**	0.65	0.48, 0.88	0.006	0.78	0.56, 1.06	0.114	0.65	0.46, 0.90	0.011
** Q4 [2.6,6.6]**	1.41	1.09, 1.83	0.010	1.66	1.26, 2.18	< 0.001	1.37	1.02, 1.83	0.037
** p for trend**			0.185			0.009			0.308
**Monocytes (continuous)**	1.71	1.01, 2.87	0.044	1.55	0.89, 2.67	0.119	1.27	0.71, 2.26	0.414
**Monocytes**									
** Q1 [0.1,0.4)**	—	—		—	—		—	—	
** Q2 [0.4,0.563)**	1.26	0.90, 1.81	0.192	1.13	0.80, 1.64	0.492	1.13	0.78, 1.66	0.527
** Q3 [0.563,0.6)**	0.02	0.00, 0.08	< 0.001	0.02	0.00, 0.09	< 0.001	0.01	0.00, 0.06	< 0.001
** Q4 [0.6,1.8]**	1.31	0.93, 1.87	0.131	1.14	0.80, 1.65	0.475	1.03	0.71, 1.52	0.874
** p for trend**			0.435			0.907			0.592
**Platelets (continuous)**	1.00	1.00, 1.00	0.504	1.00	1.00, 1.00	0.151	1.00	1.00, 1.00	0.895
**Platelets**									
** Q1 [72,214)**	—	—		—	—		—	—	
** Q2 [214,255)**	0.88	0.68, 1.14	0.332	1.00	0.77, 1.30	0.999	0.99	0.75, 1.31	0.959
** Q3 [255,287)**	0.53	0.40, 0.70	<0.001	0.65	0.49, 0.87	0.004	0.56	0.41, 0.75	<0.001
** Q4 [287,672]**	0.88	0.68, 1.13	0.312	1.20	0.92, 1.58	0.186	1.03	0.77, 1.38	0.850
** p for trend**			0.043			0.759			0.340

^1^OR = Odds Ratio, CI = Confidence Interval

Model I: no covariates were adjusted

Model II: adjusted for Age, Sex, and Race

Model III: adjusted for Shift-schedule, Age, Sex, Race, PIR, Alcohol drinking, Smoke, BMI, Self-diet report, HEI2020, Weak/failing kidneys, Stroke, Asthma, Anemia, Arthritis, Coronary heart disease, Chronic bronchitis, Cancer or malignancy, and PHQ score

These findings suggest that inflammatory factors are associated with prevalent MetS among shift workers and may be informative when assessing MetS-related risk profiles. However, these effects were attenuated after comprehensive covariate adjustments, warranting further investigation.

**Fig 6:**
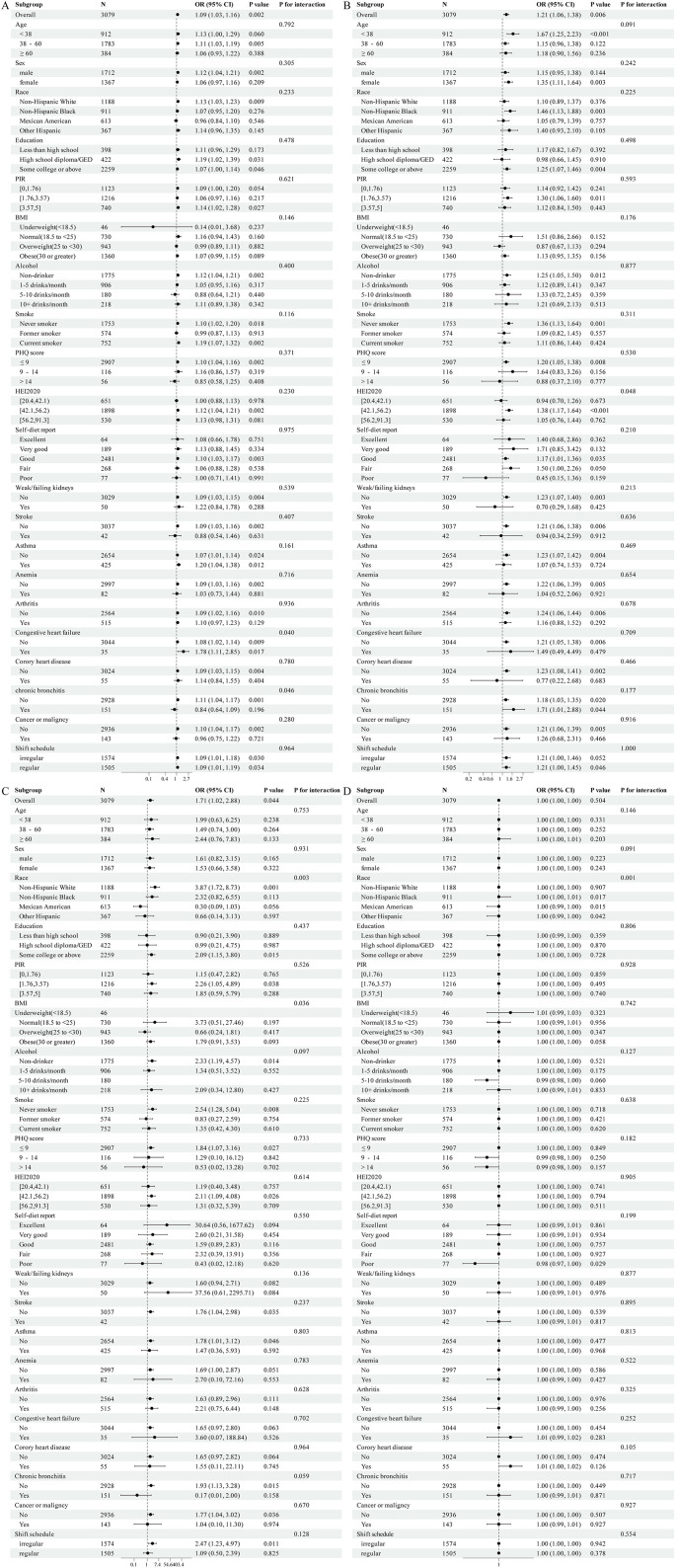
Stratified analysis of neutrophils (A), lymphocytes (B), monocytes (C) and platelets (D).

Fig 6 illustrates that neutrophils (Fig 6A), lymphocytes (Fig 6B), and monocytes (Fig 6C) were significantly associated with an increased risk of MetS (neutrophils: OR: 1.09; 95% CI: 1.03–1.16, p = 0.002, lymphocytes: OR: 1.21; 95% CI: 1.06–1.38, p = 0.006, monocytes: OR: 1.71; 95% CI: 1.02–2.88, p = 0.044). Among non-Hispanic White participants, both neutrophils (OR: 1.13; 95% CI: 1.03–1.23, p = 0.009) and monocytes (OR: 3.87; 95% CI: 1.72–-8.73, p = 0.001) demonstrated strong associations with MetS risk. Moreover, a negative correlation was observed between alcohol consumption and inflammatory factors.

### Effect of different numbers of inflammation factors on MetS

**Fig 7:**
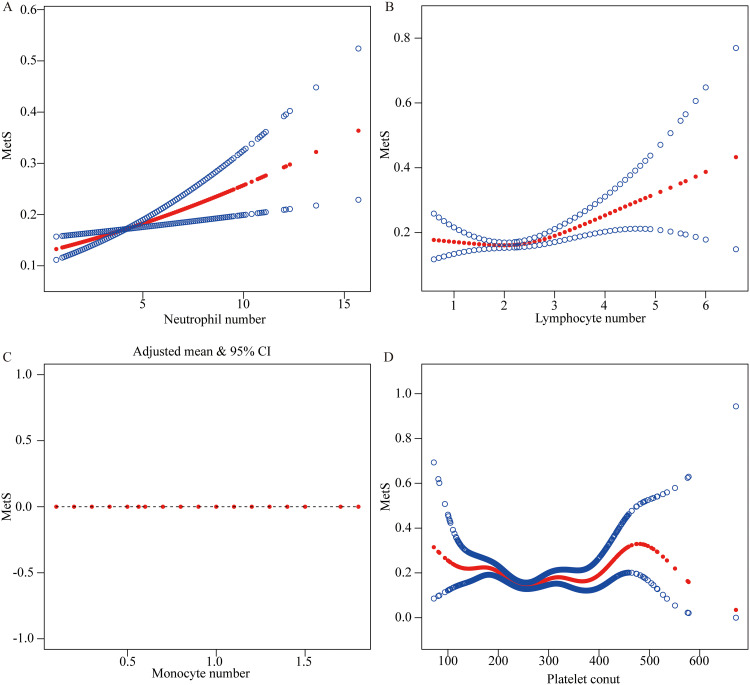
General additive models demonstrate the relationship between continuous variables (neutrophils, lymphocytes, monocytes, platelets) and MetS. The x-axis represents each continuous variable, and the y-axis shows the estimated smooth function of the log(relative risk). The central red dotted line denotes the fitted smooth effect estimated by the GAM, while the upper and lower blue dotted lines indicate the 95% confidence interval. The horizontal line at zero represents the reference level, with values above or below indicating positive or negative associations, respectively.

To better understand how variations in inflammatory factor counts influence MetS risk, we conducted a GAM analysis to identify non-linear relationships and potential threshold effects (Fig 7 and Table 3). The results indicated that the risk of MetS among shift workers increased steadily with higher neutrophil counts (Fig 7A). For lymphocytes (Fig 7B), a non-linear relationship was observed, with an inflection point at 2.2. Below this threshold, the association was inverse but not significant (OR: 0.8; 95% CI: 0.6–1.0, p = 0.083), whereas above the threshold, higher counts were significantly associated with an increased risk of MetS (OR: 1.5; 95% CI: 1.2–1.8, p < 0.001; likelihood ratio test, p = 0.002). Monocytes (Fig 7C) demonstrated a linear association with MetS risk, showing no evidence of a threshold effect. Platelet counts (Fig 7D) exhibited a more complex, S-shaped pattern, rising at low levels, plateauing between 160 and 350, and then increasing again after minor fluctuations. Two-piecewise regression identified an inflection point at 257. The effect estimates were 1.0 (95% CI: 1.0–1.0, p < 0.001) on the left and 1.0 (95% CI: 1.0–1.0, p = 0.001) on the right. Although statistically significant, the OR of 1.0 indicated no meaningful association between platelet counts and MetS. Additional analyses of other imflammatory markers are presented in S4 Table.

**Table 3:** The threshold effect analysis of monocytes and platelets on MetS.

Exposure	Neutrophils 95%CI, p-value	Lymphocytes 95%CI, p-value	Platelets 95%CI, p-value	Monocytes 95%CI, p-value
**Model I**				
Simple linear effect	1.1 (1.0, 1.2), 0.002	1.2 (1.1, 1.4), 0.006	1.0 (1.0, 1.0), 0.504	—
**Model II**				
Breakpoint (K)	4.2	2.2	257	—
Segment < K (Effect 1)	1.0 (0.9, 1.2), 0.597	0.8 (0.6, 1.0), 0.083	1.0 (1.0, 1.0), < 0.001	—
Segment > K (Effect 2)	1.1 (1.0, 1.2), 0.007	1.5 (1.2, 1.8), < 0.001	1.0 (1.0, 1.0), 0.001	—
Difference between 1 and 2	1.1 (0.9, 1.3), 0.401	2.0 (1.3, 3.0), 0.002	1.0 (1.0, 1.0), < 0.001	—
Breakpoint slope estimate	−1.6 (−1.8, −1.5)	−1.8 (−1.9, −1.6)	−1.8 (−2.0, −1.7)	—
Likelihood ratio test	0.403	0.002	< 0.001	—

Data Explanation: β (95%CI) P-value/ OR (95%CI) P-value

Outcome variable: MetS

Exposure variables: Monocytes, Platelets

Adjusted variables: Age, Weak/failing kidneys, Stroke, Asthma, PHQ Score

## Discussion

We analyzed NHANES datasets from 2005–2010 and 2017–2018 to evaluate the risk of MetS among shift workers. All four ML models (LightGBM, RF, XGBoost, and LR) demonstrated modest-to-good discriminative performance, with LightGBM achieving the highest AUC values. These findings suggest that non-linear algorithms may better capture capturing complex relationships among variables. Consistent with our findings, previous studies predicting MetS and other chronic diseases have reported that tree-based nonlinear algorithms outperform traditional linear models, particularly in handling high-dimensional data and identifying variable interactions [37,38]. For example, LightGBM and RF have been widely applied in the prediction of diabetes and cardiovascular disease, consistently showing superior performance compared with LR [39,40]. The primary objective of our study was to compare relative model performance and identify key predictors. Furthermore, through SHAP-based interpretative analysis, we found that inflammation-related markers, including lymphocytes, neutrophils, monocytes, and their derived ratios such as the NLR, MLR, SII, and SIRI, consistently ranked among the top predictors of MetS in shift workers. These findings suggest that inflammation may be closely linked with prevalent MetS in this population and may serve as a marker of adverse metabolic status.

BMI and age, as conventional risk factors, also remained among the predominant contributors. Shift work disrupts circadian rhythms, leading to irregular eating patterns that promote higher calorie intake and subsequent weight gain [41]. Furthermore, exposure to artificial light at night significantly interferes with lipid metabolism, leading to excessive hepatic fat accumulation over time [42]. Artificial light exposure may also impair sleep quality by increasing glucose and lipid absorption, reducing fatty acid release, and worsening metabolic imbalances. With aging, physiological functions decline and self-repair capacity weakens, thereby increasing susceptibility to MetS-related conditions including obesity, hyperglycemia, and hyperlipidemia. Aging also disrupts circadian rhythms [43], resulting in decreased expression of clock genes and disruption of normal biological cycles. Such circadian rhythm disorders can, in turn, accelerate aging, increase oxidative stress and inflammation, and trigger age-associated diseases including coronary heart disease and osteoarthritis [44]. Together, these mechanisms may help explain why higher BMI and older age are consistently associated with MetS.

To further investigate the impact of inflammatory factors on MetS in shift workers, we applied LR and GAM analyses. LR identified lymphocytes as relatively independent predictors, while GAM revealed both linear and non-linear associations of neutrophils, lymphocytes, monocytes, and platelets with MetS among shift workers. These findings suggest that inflammatory responses may be linked with both the presence and severity of MetS in this population and may involve threshold-like patterns that warrant further investigation.

Consistent with previous studies, alterations in shift patterns may modulate the activity of inflammatory cells through mechanisms such as circadian rhythm disruption, chronic stress, and increased metabolic load [45–47]. We observed that when lymphocyte levels exceeded approximately 2.2 × 10⁹/L, the odds of prevalent MetS were significantly higher, suggesting that lymphocytes not only reflect the degree of systemic inflammation but may also mark a potential threshold of immune activation related to MetS.

In shift workers, adipose tissue inflammation may serve as an essential pathway linking lymphocytes to metabolic dysfunction. Circadian disruption and lifestyle imbalance make long-term night shift workers particularly susceptible to obesity and insulin resistance [48]. Under these conditions, T cells and natural killer cells have been reported to accumulate in adipose tissue and release proinflammatory cytokines such as interferon-gamma and tumor necrosis factor-alpha (TNF-α), which are associated with macrophage polarization toward the M1 phenotype and impaired insulin signaling [49]. As these subsets belong to the circulating lymphocyte pool, their expansion contributes to elevated peripheral lymphocyte counts [50]. These mechanisms, described in previous experimental studies, provide a biological context that may help interpret the observed lymphocyte and MetS associations, although our cross-sectional data cannot confirm these pathways.

Circadian disruption itself constitutes a chronic stress burden, disturbing the hypothalamic-pituitary-adrenal axis and sympathetic activity, and altering cortisol and melatonin rhythms. These changes influence immune cell generation, circulation, and trafficking [51]. Lymphocytes, which are highly sensitive to circadian cues, may undergo metabolic reprogramming under prolonged night shift exposure, adopting a more proinflammatory phenotype [52,53]. Once their levels exceed a critical threshold, amplified cytokine release is associated with more pronounced adipose tissue inflammation and insulin resistance, which may be related to a higher likelihood of MetS.

We also found that neutrophil and monocyte counts were positively and approximately linearly associated with the probability of prevalent MetS. This finding does not entirely align with the conventional leukocyte distribution pattern, in which neutrophil and lymphocyte counts often show antagonistic behavior during acute inflammatory responses. One possible explanation is that the interplay between chronic metabolic disturbances and circadian disruption may alter this antagonistic relationship. According to report, Chronic inflammation in adipose tissue and metabolic dysregulation, such as fat accumulation and insulin resistance, may directly promote leukocyte proliferation [54,55]. Furthermore, circadian rhythm disruption has been shown to enhance the secretion of pro-inflammatory cytokines including interleukin (IL)-6, IL-10, IL-17, and TNF-α, thereby exacerbating leukocyte activation and contributing to the concurrent elevation of neutrophil and monocyte counts [55,56]. In contrast, platelets were not significantly associated with MetS, although previous studies has reported associations between platelet-related indices and metabolic risk [57]. Circadian disruption, common among shift workers, can alter platelet production and activity by affecting the bone marrow microenvironment and through stress-related sympathetic and hormonal pathways, potentially promoting proinflammatory and procoagulant states [58,59]. However, the observed decrease in SHAP values for neutrophils within the 10–12 count range may have been influenced by the relatively sparse sample distribution in this interval, which could affect the robustness of this result.

In this study, composite inflammatory indices, including MLR, NMLR, NLR, PLR, SII, SIRI, and AISI showed relatively strong associations with MetS and frequently ranked among the top 15 features in importance analyses. Notably, all these indices are closely associated with lymphocyte counts. Unlike single-cell measures, these indices integrate data from multiple immune cell types and may more accurately reflect the balance between pro- and anti-inflammatory processes. Prior studies have similarly reported that MLR, NMLR, NLR, PLR, SII, SIRI, and AISI are associated with metabolic disorders and cardiovascular events and can improve risk stratification [60,61]. Jiang et al. demonstrated that SII and SIRI independently predicted MetS, and their levels were positively correlated with obesity severity in individuals with obesity [62,63]. Mechanistically, these composite indices may more effectively capture the interplay between innate and adaptive immunity, enhancing sensitivity to early inflammatory disturbances that precede the onset of metabolic abnormalities.

Importantly, racial differences were observed. Among non-Hispanic White participants, both neutrophils and monocytes exhibited stronger associations with MetS risk than the overall population, with the effect particularly pronounced for monocytes (OR: 3.87). In contrast, lymphocytes showed a stronger association among non-Hispanic Black participants (OR: 1.46, p = 0.003). These findings suggest that inflammatory pathways may exert population-specific effects, potentially influenced by genetic background, dietary patterns, lifestyle behaviours, and socioeconomic conditions [64,65]. Such ethnic heterogeneity highlights the need for tailored predictive models and targeted interventions to more accurately account for differential inflammatory responses across diverse populations.

This study has several limitations. First, the study population was primarily derived from the United States, which may limit the generalizability of the findings to other regions and ethnic groups. Additional research in multi-ethnic and multinational cohorts is warranted to assess the applicability of these results. Second, despite adjustment for a wide range of covariates, residual confounding from unmeasured variables cannot be excluded. Several important factors, such as sleep quality, psychological stress, and occupational category (e.g., healthcare vs. industrial shift workers), were not available in the dataset. These unmeasured variables may be related to both inflammatory status and metabolic outcomes. Advanced statistical methods and longitudinal study designs will be necessary to better address potential bias. Moreover, future studies directly comparing shift workers and day workers would help clarify how shift work is linked to metabolic risk. From a modeling perspective, the four ML algorithms achieved only modest discrimination in the held-out test set and were not externally validated, so generalizability of the prediction models to other shift-working populations remains uncertain. In addition, although NHANES sampling weights and complex survey design were applied in descriptive and regression analyses, the ML models were fit without survey weighting and are best regarded as internal, sample-based tools for benchmarking algorithms and exploring predictors associated with MetS rather than definitive population-level risk prediction tools.

## Conclusion

We applied ML techniques to identify key risk factors for MetS in shift workers. Among the tested models, the RF model demonstrated the highest discriminative performance. Our analysis revealed that inflammatory biomarkers, including neutrophils, lymphocytes, monocytes, and platelets, were particularly effective in predicting MetS risk, with lymphocytes showing the strongest correlation. Further investigation of threshold values showed that lymphocyte levels exceeding approximately 2.2 × 10^9^/L were closely associated with MetS, suggesting that even slight elevations above this threshold could be clinically significant. Overall, these findings suggest that lymphocyte counts and composite inflammatory indices may be useful markers for early odds-based stratification of MetS among shift workers, whereas the extent to which monitoring or modifying these markers is associated with changes in MetS odds will need to be clarified in longitudinal and interventional studies.

## Supporting information

S1 Table Performance metrics of machine learning models prediction on the training dataset. (PDF)

S2 Table Performance metrics of machine learning models prediction on the training dataset. (PDF)

S3 Table Multivariate logistic regression analysis of different inflammation factors. (PDF)

S4 Table The threshold effect analysis of Neutrophils, Lymphocytes, Platelets and monocytes on MetS. (PDF)

## Abbreviations

AUC: Under the curve
ACC: Accuracy
AISI: Systemic inflammatory composite index
BMI: Body mass index
CBC: Complete blood count
CI: Confidence interval
GAM: Generalized additive model
GED: General educational development
HEI2020: Healthy eating index-2020
IL-6: Interleukin 6
LDL: Low-density lipoprotein
LightGBM: Light gradient boosting machine
LR: Linear regression
MEC: Mobile examination center
ML: Machine learning
MLR: Monocyte-to-lymphocyte ratio
NHANES: National Health and Nutrition Examination Survey
NLR: Neutrophil-to-lymphocyte ratio
NMLR: Neutrophil-to-monocyte ratio
OR: Odds ratios
PHQ: Patient health questionnaire
PIR: Poverty-income ratio
PLR: Platelet-to-lymphocyte ratio
RF: Random forest
ROC: Receiver operating characteristic
SHAP: SHapley Additive exPlanations
SII: Systemic immune-inflammation index
SIRI: Systemic inflammatory response index
TNF-α: Tumor necrosis factor alpha
XGBoost: eXtreme Gradient Boosting
