# Supplementary material for: Lymphocytes and related inflammatory factors as predictors of metabolic syndrome risk in shift workers: A machine learning approach based on large-scale population data
Source: PLoS One. 2025 Dec 29;20(12):e0339673. doi: 10.1371/journal.pone.0339673 (PMC12747363; doi:10.1371/journal.pone.0339673)
Supplement: S1 Table — (PDF) [file pone.0339673.s001.pdf]

Table S1. Performance metrics of machine learning models prediction on the training dataset.

Model	AUC95% CI	Accuracy	PPV (Precision)	NPV	Recall (Sensitivity)	Specificity	F1-score
RF	0.741 (0.710-0.759)	0.819	0.353	0.822	0.016	0.994	0.298
LightGBM	0.944 (0.930-0.958)	0.907	0.691	0.963	0.827	0.923	0.753
XGBoost	0.825 (0.804-0.846)	0.827	0.000	0.828	0.000	0.999	—
LR	0.763 (0.739-0.788)	0.639	0.294	0.931	0.782	0.609	0.427
