# Supplementary material for: Lymphocytes and related inflammatory factors as predictors of metabolic syndrome risk in shift workers: A machine learning approach based on large-scale population data
Source: PLoS One. 2025 Dec 29;20(12):e0339673. doi: 10.1371/journal.pone.0339673 (PMC12747363; doi:10.1371/journal.pone.0339673)
Supplement: S2 Table — (PDF) [file pone.0339673.s002.pdf]

Table S2. Performance metrics of machine learning models prediction on the test dataset.

Model	AUC95% CI	Accuracy	PPV (Precision)	NPV	Recall (Sensitivity)	Specificity	F1-score
RF	0.729 (0.699–0.777)	0.847	0.625	0.849	0.035	0.996	0.067
LightGBM	0.722 (0.682–0.762)	0.236	0.660	0.124	—	0.053	—
XGBoost	0.743 (0.705–0.781)	—	1.000	0.171	—	0.094	—
LR	0.699 (0.657–0.741)	0.162	0.753	0.100	—	0.028	—
