# Supplementary material for: Lymphocytes and related inflammatory factors as predictors of metabolic syndrome risk in shift workers: A machine learning approach based on large-scale population data
Source: PLoS One. 2025 Dec 29;20(12):e0339673. doi: 10.1371/journal.pone.0339673 (PMC12747363; doi:10.1371/journal.pone.0339673)
Supplement: S3 Table — (PDF) [file pone.0339673.s003.pdf]

Table S3. Multivariate logistic regression analysis of different inflammation factors.

Characteristic	Model I			Model II			Model III		
	OR[1]	95% CI[1]	p-value	OR[1]	95% CI[1]	p-value	OR[1]	95% CI[1]	p-value
**SII (continuous)**	1.00	1.00, 1.00	0.505	1.00	1.00, 1.00	0.358	1.00	1.00, 1.00	0.947
**SII**									
Q1	—	—		—	—		—	—	
Q2	0.95	0.74, 1.21	0.666	0.94	0.72, 1.21	0.616	0.81	0.62, 1.07	0.140
Q3	0.61	0.47, 0.80	<0.001	0.64	0.49, 0.85	0.002	0.47	0.35, 0.63	<0.001
Q4	1.01	0.79, 1.29	0.950	1.07	0.83, 1.39	0.608	0.85	0.64, 1.12	0.246
P for trend			0.376			0.762			0.034
**SIRI (continuous)**	1.12	1.00, 1.25	0.043	1.09	0.97, 1.22	0.154	1.07	0.93, 1.22	0.319
**SIRI**									
Q1	—	—		—	—		—	—	
Q2	0.99	0.77, 1.27	0.941	0.90	0.69, 1.17	0.430	0.84	0.64, 1.11	0.213
Q3	0.67	0.51, 0.88	0.004	0.63	0.47, 0.83	0.001	0.48	0.35, 0.64	<0.001
Q4	1.10	0.86, 1.41	0.440	1.00	0.76, 1.30	0.977	0.84	0.63, 1.11	0.214
P for trend			0.931			0.482			0.033
**AISI (continuous)**	1.00	1.00, 1.00	0.077	1.00	1.00, 1.00	0.060	1.00	1.00, 1.00	0.340
**AISI**									
Q1	—	—		—	—		—	—	
Q2	1.20	0.94, 1.54	0.146	1.12	0.87, 1.46	0.375	0.99	0.76, 1.31	0.967
Q3	0.55	0.41, 0.73	<0.001	0.56	0.42, 0.76	<0.001	0.41	0.30, 0.56	<0.001
Q4	1.24	0.97, 1.59	0.081	1.28	0.99, 1.67	0.060	1.03	0.78, 1.36	0.852

P for trend			0.951			0.668			0.188
**MLR (continuous)**	1.06	0.43, 2.46	0.903	0.55	0.21, 1.40	0.222	0.87	0.31, 2.37	0.793
**MLR**									
Q1	—	—		—	—		—	—	
Q2	0.86	0.67, 1.10	0.232	0.81	0.63, 1.04	0.104	0.87	0.67, 1.14	0.321
Q3	0.54	0.41, 0.71	<0.001	0.50	0.37, 0.66	<0.001	0.46	0.34, 0.62	<0.001
Q4	0.83	0.65, 1.07	0.150	0.68	0.52, 0.89	0.005	0.75	0.56, 0.99	0.044
P for trend			0.019			<0.001			0.002
**NLR (continuous)**	1.05	0.96, 1.15	0.290	1.01	0.92, 1.11	0.816	0.99	0.89, 1.10	0.872
**NLR**									
Q1	—	—		—	—		—	—	
Q2	0.95	0.74, 1.22	0.695	0.85	0.65, 1.11	0.228	0.81	0.61, 1.07	0.133
Q3	0.64	0.49, 0.84	0.002	0.65	0.49, 0.86	0.003	0.51	0.38, 0.68	<0.001
Q4	1.11	0.87, 1.42	0.382	0.96	0.74, 1.25	0.784	0.84	0.64, 1.12	0.233
P for trend			0.958			0.475			0.056
**NMLR (continuous)**	1.04	0.96, 1.13	0.313	1.00	0.91, 1.10	0.923	0.99	0.89, 1.09	0.859
**NMLR**									
Q1	—	—		—	—		—	—	
Q2	0.91	0.71, 1.16	0.439	0.79	0.61, 1.03	0.080	0.75	0.57, 0.99	0.043
Q3	0.63	0.48, 0.82	<0.001	0.62	0.46, 0.81	<0.001	0.48	0.36, 0.65	<0.001
Q4	1.06	0.83, 1.35	0.643	0.89	0.69, 1.16	0.405	0.79	0.60, 1.05	0.100
P for trend			0.743			0.225			0.021
**PLR (continuous)**	1.00	1.00, 1.00	0.034	1.00	1.00, 1.00	0.087	1.00	1.00, 1.00	0.229

**PLR**									
Q1	—	—		—	—		—	—	
Q2	0.74	0.58, 0.94	0.015	0.83	0.64, 1.07	0.148	0.82	0.63, 1.07	0.145
Q3	0.52	0.40, 0.67	<0.001	0.58	0.44, 0.76	<0.001	0.51	0.38, 0.68	<0.001
Q4	0.70	0.55, 0.89	0.004	0.73	0.57, 0.94	0.016	0.78	0.59, 1.02	0.069
P for trend			<0.001			0.002			0.004

[1]OR = Odds Ratio, CI = Confidence Interval

Model I: no covariates were adjusted

Model II: adjusted for Age, Sex, and Race

Model III: adjusted for Shift-schedule, Age, Sex, Race, PIR, Alcohol drinking, Smoke, BMI, Self-diet report, HEI2020, Weak/failing kidneys, Stroke, Asthma, Anemia, Arthritis, Coronary heart disease, Chronic bronchitis, Cancer or malignancy, and PHQ score
