# Supplementary material for: Lymphocytes and related inflammatory factors as predictors of metabolic syndrome risk in shift workers: A machine learning approach based on large-scale population data
Source: PLoS One. 2025 Dec 29;20(12):e0339673. doi: 10.1371/journal.pone.0339673 (PMC12747363; doi:10.1371/journal.pone.0339673)
Supplement: S4 Table — (PDF) [file pone.0339673.s004.pdf]

Table S4. The threshold effect analysis of Neutrophils, Lymphocytes, Platelets and monocytes on MetS.

Exposure	SII	SIRI	AISI	MLR	NMLR	NLR	PLR
**Model I**							
Simple linear effect	1.0 (1.0, 1.0) 0.565	1.1 (1.0, 1.3) 0.019	1.0 (1.0, 1.0) 0.059	1.2 (0.5, 2.9) 0.698	1.1 (1.0, 1.2) 0.234	1.1 (1.0, 1.2) 0.220	1.0 (1.0, 1.0) 0.029
**Model II**							
Breakpoint (K)	516.3	1.1	300	0.3	2.3	2	123.5
Segment < K (Effect 1)	1.0 (1.0, 1.0) 0.016	0.7 (0.5, 1.0) 0.085	1.0 (1.0, 1.0) 0.008	0.0 (0.0, 0.3) <0.001	0.8 (0.6, 1.0) 0.022	0.8 (0.6, 1.0) 0.030	1.0 (1.0, 1.0) <0.001
Segment > K (Effect 2)	1.0 (1.0, 1.0) 0.019	1.3 (1.1, 1.5) <0.001	1.0 (1.0, 1.0) <0.001	9.8 (2.7, 35.8) <0.001	1.2 (1.1, 1.3) 0.003	1.2 (1.1, 1.3) 0.004	1.0 (1.0, 1.0) 0.023
Difference between 1 and 2	1.0 (1.0, 1.0) 0.005	1.8 (1.1, 2.8) 0.010	1.0 (1.0, 1.0) <0.001	204.8 (15.7, 2667.8) <0.001	1.5 (1.2, 2.1) 0.003	1.6 (1.1, 2.1) 0.005	1.0 (1.0, 1.0) <0.001
Breakpoint slope estimate	-1.7 (-1.9, -1.6)	-1.7 (-1.9, -1.6)	-1.8 (-1.9, -1.6)	-1.8 (-2.0, -1.7)	-1.7 (-1.9, -1.6)	-1.7 (-1.9, -1.6)	-1.9 (-2.0, -1.7)
Likelihood ratio test	0.005	0.011	<0.001	<0.001	0.003	0.005	<0.001

Data Explanation: β (95%CI) P-value / OR (95%CI) P-value
Outcome variable: MetS
Exposure variables: Monocytes, Platelets
